# Does 1,8-diiodooctane affect the aggregation state of PC_71_BM in solution?

**DOI:** 10.1098/rsos.180937

**Published:** 2018-09-12

**Authors:** Gabriel Bernardo, Adam. L. Washington, Yiwei Zhang, Stephen. M. King, Daniel. T. W. Toolan, Michael P. Weir, Alan D. F. Dunbar, Jonathan R. Howse, Rajeev Dattani, John Patrick A. Fairclough, Andrew J. Parnell

**Affiliations:** 1 Department of Physics and Astronomy, University of Sheffield, Sheffield S3 7RH, UK; 2 LEPABE, Department of Chemical Engineering, University of Porto, 4200-465 Porto, Portugal; 3 ISIS Pulsed Neutron and Muon Source, Science and Technology Facilities Council, Rutherford Appleton Laboratory, Harwell Campus, Didcot OX11 0QX, UK; 4 Department of Mechanical Engineering, University of Sheffield, Sheffield S3 7HQ, UK; 5 Department of Chemical and Biological Engineering, University of Sheffield, Sheffield S1 3JD, UK; 6 European Synchrotron Radiation Facility (ESRF), Grenoble, France

**Keywords:** polymer solar cell, fullerene, SAXS, SANS, morphology

## Abstract

1,8-Diiodooctane (DIO) is an additive used in the processing of organic photovoltaics and has previously been reported, on the basis of small-angle X-ray scattering (SAXS) measurements, to deflocculate nano-aggregates of [6,6]-phenyl-C71-butyric acid methyl ester (PC_71_BM) in chlorobenzene. We have critically re-examined this finding in a series of scattering measurements using both X-rays and neutrons. With SAXS, we find that the form of the background solvent scattering is influenced by the presence of DIO, that there is substantial attenuation of the X-rays by the background solvent and that there appears to be beam-induced aggregation. All three factors call into question the suitability of SAXS for measurements on these samples. By contrast, small-angle neutron scattering (SANS) measurements, performed at concentrations of 15 mg ml^−1^ up to and including 40 mg ml^−1^, show no difference in the aggregation state for PC_71_BM in chlorobenzene with and without 3% DIO; we find PC_71_BM to be molecularly dissolved in all solvent cases. *In* *situ* film thinning measurements of spin-coated PC_71_BM solution with the DIO additive dry much slower. Optical imaging shows that the fullerene films possess enhanced molecular mobility in the presence of DIO and it is this which, we conclude, improves the nanomorphology and consequently solar cell performance. We propose that any compatible high boiling solvent would be expected to show the same behaviour.

## 1. Introduction

The addition of 1,8-diiodooctane (DIO) [1,2] (and other high boiling point additives [3]) to polymer–fullerene blends has a large influence on organic photovoltaic device performance and its use has enabled some of the highest efficiency devices observed at the time of writing [4]. Even now, as device scientists and engineers shift their focus to non-fullerene blend systems, the use of DIO in the casting solvent still persists and has very recently enabled a PCE over 13% to be achieved in a single junction organic solar cell [5]. There is, however, some ongoing debate in the field as to the exact role of DIO [6–11]. In particular, debate exists around its influence on the solution state of fullerenes such as [6,6]-phenyl-C71-butyric acid methyl ester (PC_71_BM) [12,13]. The primary role of DIO is to selectively dissolve one of the components of a polymer solar cell blend such that the film takes longer to form than it otherwise would with just a conventional solvent such as chlorobenzene. This extension in the drying time allows the bulk heterojunction nanostructure to evolve to give the appropriate length scales for solar cell device operation. A second suggested mode of action is to reduce the size of fullerene aggregates in solution prior to spin coating, as these could be detrimental to device performance by reducing the amount of interface within the device capable of extracting useful excitons. This second mechanism was proposed by Lou *et al.* [14] who examined the solution structure of PC_71_BM in chlorobenzene (CB) using small-angle X-ray scattering (SAXS) and found that the addition of a small amount of DIO (3% v/v) to CB altered the scattering signal of the fullerene solution. Their model-based analysis of the scattering data led them to interpret this as evidence that DIO improves the solubility of PC_71_BM when mixed with chlorobenzene. This reduction in the PC_71_BM aggregate size in solution was hypothesized to be the reason for improvement in OPV device efficiencies, when spin-coated with the polymer PTB7, as a result of improved intercalation of the fullerene. One of their main conclusions was that ‘While DIO molecules cause only slight changes in the size of the PTB7 aggregates in solution, they selectively and completely dissolve the PC_71_BM aggregates’ [14]. However, Das *et al.* [15] used small-angle neutron scattering (SANS) to study the solution state of PC_71_BM in pure dichlorobenzene (DCB) and in DCB with 3 wt% DIO. They concluded that there was no difference in the solution state of the PC_71_BM in DCB solution and that it was molecularly dissolved in both instances. In the light of contrary evidence, we set out to understand how DIO alters the solution behaviour of fullerenes in a simple fullerene solvent system and so decided to repeat these measurements. We have recently examined the drying behaviour of polymer blend devices using DIO and observed markedly different drying times for films with and without DIO [16]. We concluded [16], as had others [17], that nanostructure evolution during film drying was the main effect of adding DIO, but aimed to understand the action of DIO on fullerenes in solution more generally also.

## 2. Material and methods

**Figure 1.**
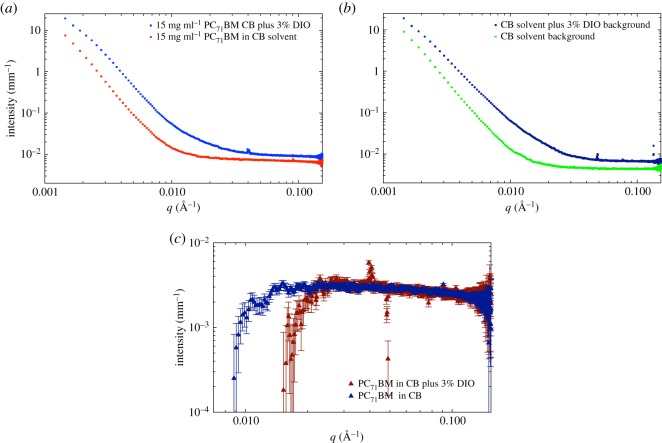
SAXS data for PC_71_BM (95% purity) at a concentration of 15 mg ml^−1^ in pure chlorobenzene and chlorobenzene with 3% v/v DIO (*a*) without background subtraction. (*b*) Scattering from the two respective background solvents, showing the large difference. (*c*) The solvent-subtracted SAXS data for the two cases of PC_71_BM with and without 3% v/v DIO.

**Figure 2.**
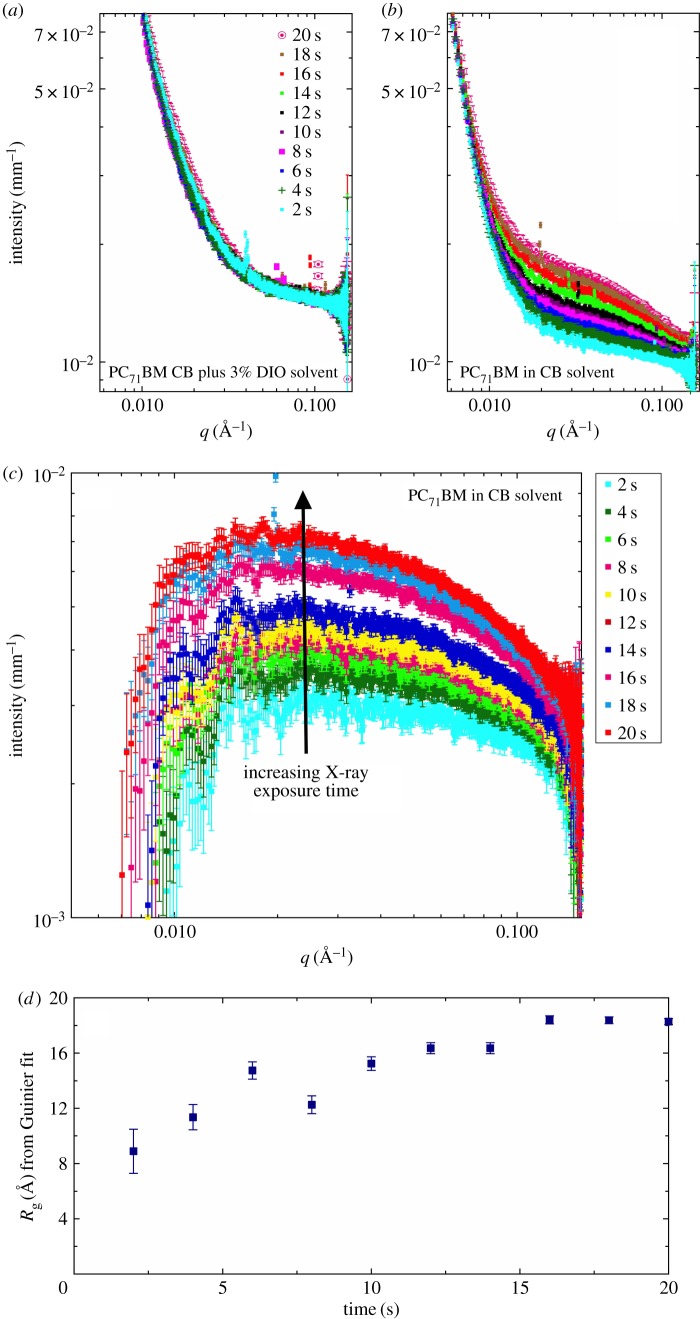
SAXS solution data for PC_71_BM (95% purity) solutions at a concentration of 15 mg ml^−1^ in (*a*) chlorobenzene with 3% v/v DIO and in (*b*) pure chlorobenzene. The result of solvent subtraction for (*b*) is shown in (*c*), and in (*d*), the shape-independent Guinier model-derived *R*_g_ as a function of cumulative SAXS exposure time.

**Figure 3.**
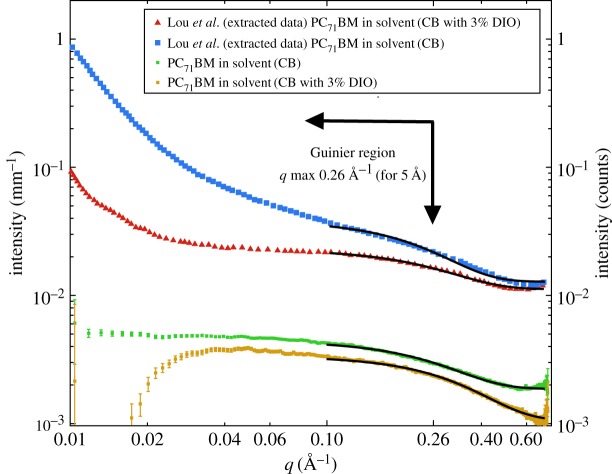
SAXS solution data for PC_71_BM at a concentration of 15 mg ml^−1^ in pure chlorobenzene and chlorobenzene with 3% v/v DIO. The data from the Lou *et al.* [14] paper have been extracted using GraphClick 3.0 to enable comparison with our own SAXS data.

**Figure 4.**
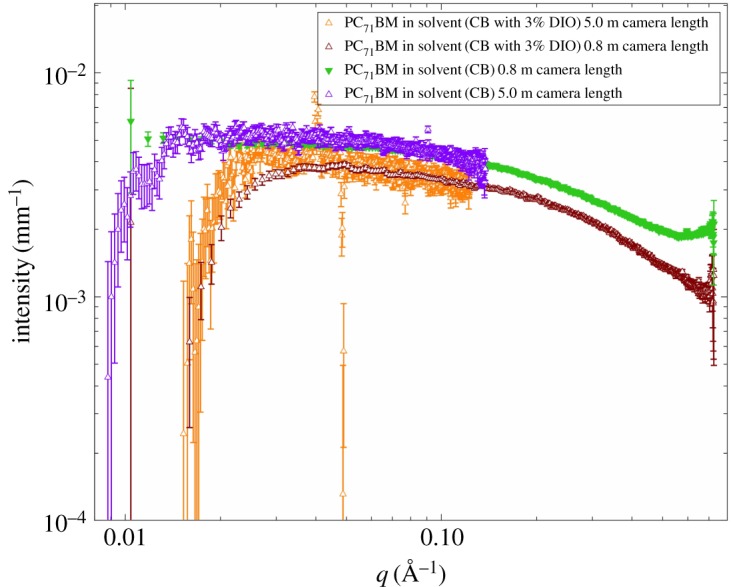
SAXS solution data for PC_71_BM at a concentration of 15 mg ml^−1^ in pure chlorobenzene, chlorobenzene with 3% v/v DIO for both camera distances (5 and 0.8 m) used in the SAXS measurements showing that the shape and overlap are consistent between the two measured *q* ranges used.

**Figure 5.**
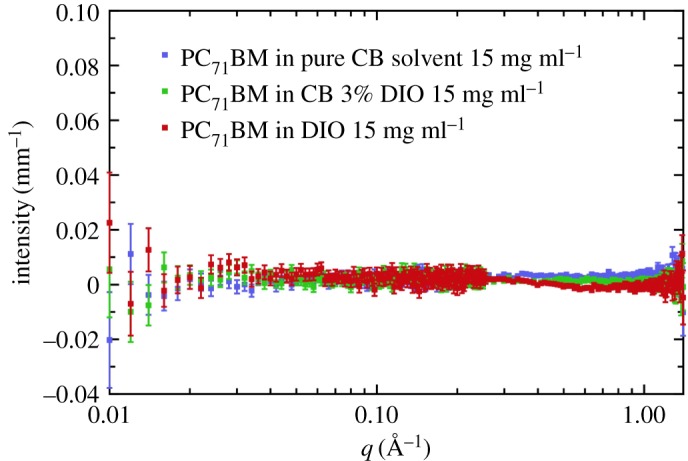
SANS solution data for PC_71_BM at a concentration of 15 mg ml^−1^ in pure chlorobenzene, chlorobenzene with 3% v/v DIO, and also in pure DIO.

**Figure 6.**
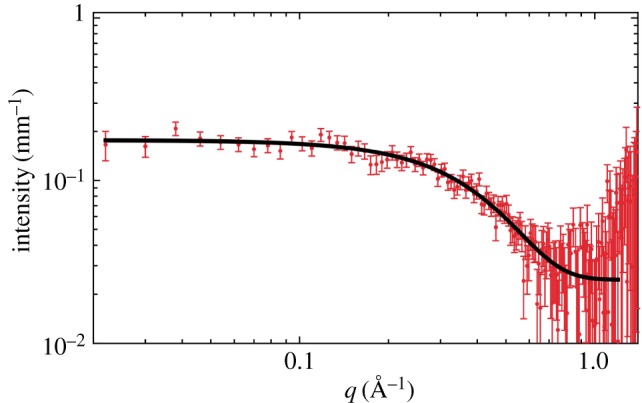
SANS solution data for PC_71_BM at a concentration of 15 mg ml^−1^ in pure carbon disulfide (CS_2_).

For the SANS data presented in figures 5 and 6, we used 99% purity PC_71_BM. While for the SAXS data (figures 1–4; electronic supplementary material, figure S3) where we measured higher concentration solutions of PC_71_BM (40 mg ml^−1^), we used 95% purity PC_71_BM. All of the fullerene materials were purchased from Ossila, UK and are of high quality as evidenced by our ability to produce high-efficiency devices [16]. The solutions used in the SAXS and SANS experiments described here were prepared by dissolving the materials in chlorobenzene (99% purity) with/without 3% by volume DIO (98% purity), both purchased from Sigma–Aldrich.

SAXS measurements were performed on the beamline ID02 at the ESRF (Grenoble, France) using an X-ray energy of 12.47 keV [18]. Samples were mounted in quartz capillaries nominally of 1.5 mm inner diameter (Capillary Tube Supplies Ltd, UK), though there is known to be some variation in the capillary diameter. To compensate for this, each capillary was individually measured and that diameter value was used to correct the data for path length. We had a measurement time of 2 s per frame. As we did not perform the experiment under flow, a fast shutter was employed with a closing time of 1.3 ms, to reduce the exposure of the solution over long periods (minutes) and to allow us to assess for beam damage. The X-ray beam spot size was 250 × 250 µm. The sample-to-detector distance was 5.0 m in figures 1 and 2 and 0.8 m in figure 3, and the same quartz capillaries and solutions were used for both sets of measurements. Data were recorded on a Rayonix MX-170HS detector, then normalized for transmission and reduced using the program SAXSutilities (http://www.sztucki.de/index_en.php) developed by Michael Sztucki (principally using the modules EDF plot and data tools to mask the beamstop and then perform radial integration) to give the one-dimensional scattering data, intensity versus *q* (where *q* = (4π/*λ*) sin*θ*, *λ* is the wavelength and 2*θ* is the scattering angle).

Further SAXS data were taken in a flow capillary geometry using a borosilicate glass capillary (Capillary Tube Supplies Ltd, UK) with a diameter of approximately 0.5 mm. This made possible measurements with a SAXS laboratory beamline using a liquid gallium MetalJet (Excillum) X-ray source (9.2 keV, *λ* = 1.34 Å). The scattered X-rays were detected using a Pilatus3 R 1M detector. The data were processed using Foxtrot software and scaling the data for transmission and thickness. Solvent subtractions were then performed using SASView.

SANS measurements were performed on the LOQ time-of-flight diffractometer at the ISIS Pulsed Neutron Source (Didcot, UK) [19]. The sample-to-detector distance was 4.1 m. Solutions were pipetted into PTFE-stoppered 1 mm pathlength quartz cuvettes (Hellma UK) that were mounted on a computer-controlled sample changer maintained at 25°C and data were recorded for approximately 90 min sample^−1^. The neutron beam diameter was 12 mm. Each raw scattering dataset was corrected for the incident neutron wavelength distribution, detector efficiency and spatial linearity, the measured transmission and cuvette pathlength, before being radially averaged and converted to coherent elastic scattering cross-section data (∂*Σ*/∂*Ω*) as a function of the scattering vector, *q*, using the Mantid framework [20]. Here, for convenience, we follow the normal convention of referring to ∂*Σ*/∂*Ω* as intensity, *I*. These reduced data were placed on an absolute scale by scaling to the scattering from a partially deuterated polymer blend of known molecular weight (which determines *I*(0)) measured under identical conditions, in accordance with established procedures [21].

The reduced SAXS or SANS from an empty capillary or cuvette, respectively, was then subtracted to yield data free of any scattering contribution from either the sample containers or the instrument. Volume fraction-adjusted proportions of the resulting solvent scattering data were then subtracted from the equivalent solution data.

The SAXS data from Lou *et al.* [14] (appearing in fig. 1*b* of that article) depicted in figure 3 here were extracted using the software GraphClick 3.0 (Arizona Software).

To enable the comparison of the relative scattering strength, we have calculated the scattering length densities for different X-ray energies and for neutrons. These were calculated using the NIST activation and scattering calculator (https://www.ncnr.nist.gov/resources/activation/).

For the *in situ* interferometric measurements, the substrates were single crystal silicon (Prolog Semicor Ukraine) spin-coated with a thin PEDOT : PSS (Clevios AI 4083) layer, 54 nm in thickness. The PC_71_BM was spin cast at a spin speed of 1500 r.p.m. Measurements of the film thinning dynamics were performed using the stroboscopic interference microscope set-up described in detail elsewhere [22,23]. Briefly, a small DC motor acts as a spin-coater, which is mounted directly under a 10× microscope objective. The current supply to the motor shows sharp dips as the brushes pass between the commutators and this is used to trigger both a 50 µs light pulse from a 620 nm LED (Luxeon Rebel Red) and image collection once per revolution. In our experimental geometry, the Bragg equation for constructive interference simplifies to *d* = *mλ*/3 (approximating polymer/solvent refractive indices to 1.5), where *m* is an integer value. Using near monochromatic illumination (*λ* = 620 nm, Δ*λ* = 20 nm FWHM), constructive interference occurs as the film thins through every 206.67 nm. Drying curves were obtained through the application of an automated LabView fringe counting algorithm to a selected region across the field of view of the collected image sequences. These drying curves were placed on an absolute scale by adjusting their final end height to correspond to an area-averaged thickness of the final films obtained through ellipsometry.

The scanning probe microscopy measurements were carried out using a Dimension 3100 microscope in a tapping mode to measure the surface topography of the spin-coated films. The tapping tips had a resonance near 275 kHz.

## 3. Results and discussion

Solubility measurements have shown that PC_71_BM has an experimentally measured solubility limit of up to 210 mg ml^−1^ in chlorobenzene [24]. Consequently, it is reasonable to expect the fullerene to be molecularly dissolved in chlorobenzene solution at a concentration of 15 mg ml^−1^, given adequate preparation time. However, Lou *et al.* attributed the benefits of adding DIO to such a solution as promoting the deflocculation of PC_71_BM aggregates.

We performed a series of similar SAXS measurements on fullerene blends dissolved in halogenated solvents at an X-ray energy of 12.47 keV, very similar to the actual X-ray energy (13 keV) used by Lou *et al.* [14] (their paper incorrectly states 7 keV and has since been amended online; L. Chen 2017, personal communication). Our total *q* range (0.0015–0.7 Å^−1^) covers the entirety of the *q*-window studied in Lou *et al.*'s work [14] (0.01–0.7 Å^−1^; length scales of 628–9 Å), with our measurements also including larger structures out to 4188 Å.

Our SAXS data shown in figure 1 have been corrected for sample transmission and capillary thickness. The SAXS data prior to background subtraction are shown in figure 1*a*,*b* with and without DIO, respectively, while the data after solvent subtraction are shown in figure 1*c*. The first important point to note is that these are weakly scattering samples, that is, a small signal relative to the background solvent scatter. In figure 1*b*, we have plotted the respective solvents with and without the addition of DIO, observing a significant factor of 1.5 difference in the intensity of the background between the two solvents. However, more importantly, it is clear that the *q*-dependence of the scattering at *q* < 0.02 Å^−1^ is different for the two solvents. In other words, the intermediate-range molecular order in the two solvent systems (CB only and CB with DIO) appears to be different. In figure 1*c*, we plot the respective solvent-subtracted data for the case of PC_71_BM with and without DIO. This shows flat, featureless scattering, which diverges below a certain *q* value. This divergence is due to the over-subtraction of the background signal and probably arises for a number of reasons, including variations in the actual pathlength of the capillaries we used (1.5 mm), variations in the wall thickness of the capillaries (nominally 0.01 mm) and uncertainties in the calculation of the correct proportion of solvent scattering to subtract. Lou *et al.* [14] used a 1.5 mm pathlength flow cell which would have circumvented issues with pathlength variations.

In electronic supplementary material, figure S1, we plot the transmission values for all the solutions we have measured using SAXS. It can be seen that these values are very low because of the high electron density of both the Cl atoms, and especially the I atoms, which significantly attenuate the X-ray beam (the attenuation length of DIO—the pathlength needed to stop 1/*e* of the incoming beam is 89 µm at 12.47 keV and 100 µm at 13 keV; these values are about one-third that of aluminium), and in part because of the sample path lengths. The Beer–Lambert law tells us that thicker samples will have lower transmissions. Low transmission values can be indicative of multiple scattering effects, but also mean that a significant proportion of the energy of the incoming X-ray beam is being absorbed by the sample. The effect of so much energy flux being absorbed in such a small volume is likely to mean that the measurement cannot be assumed to be a passive one. There is a high probability that the beam may, in fact, induce aggregation and clustering of individual particles due to the photoelectrons released upon X-ray absorption [25]. To test this, in figure 2, we show the effect of exposure time (on successive frames) on the measured SAXS signal. With increasing X-ray exposure, the sample without DIO (figure 2*b*) displays an increase in the SAXS signal. This is not seen in the solvent (electronic supplementary material, figure S2) and so must come from the PC_71_BM, presumably due to X-ray-induced aggregation. Interestingly, this effect is not seen in the sample containing DIO in figure 2*a*.

To confirm our initial speculation that the X-ray beam is causing beam-induced aggregation of PC_71_BM, we fitted the SAXS data to a Guinier function over the range 0.02 Å^−1^ < *q* < 0.15 Å^−1^ using SasView 4.1 to obtain an effective *R*_g_. These *R*_g_ values are shown in figure 2*d* as a function of time up to 20 s total beam exposure. Over this period, the *R*_g_ has more than doubled from 8 to 18 Å.

We also examined the high *q* data (*q* > 0.1 Å^−1^) taken at a camera length of 0.8 m, to more accurately look for differences in the *R*_g_ of the fullerene with and without the addition of DIO. The data are plotted in figure 3. What is clear is that our solution data have a reasonable Guinier plateau for both solvent cases, with minimal scattering at low *q*; this agrees well with our longer camera length SAXS data, which also plateaus in this *q* range. Fitting the data to a Guinier model for the region *q* = 0.1 Å^−1^ and above, we find, for the sample in CB only, that *R*_g_ is 6.1 ± 0.02 Å, and for the sample in CB with DIO *R*_g_ is 4.9 ± 0.02 Å. Performing the same fit to the extracted data of Lou *et al.* yields *R*_g_ values of 6.8 and 6.0 Å, respectively (but we obviously cannot estimate the uncertainties). These latter values are not dissimilar to our own, and rather less than the 11.5–12.6 Å reported by Lou *et al.*, who chose to fit more complex multi-level empirical models for the case without DIO [14]. The overestimation of the *R*_g_ with Lou *et al*'s use of these two models may be an artefact of incorrectly fitting the low-*q* data first in a multi-level model such as the unified fit, where the model should, by definition, be assembled by fitting the highest-*q* (smallest structural level) first and then progressing sequentially to larger and larger (lower-*q*) structures [26,27].

To further validate the Guinier plateau seen in the high *q* data (*q* > 0.1 Å^−1^) measured with a 0.8 m camera distance, we have plotted the data collected at both camera distances (5.0 and 0.8 m) in figure 4, where the shape and overlap are seen to be consistent. The presence of a Guinier plateau confirms that these are indeed isolated scatterers, with *R*_g_ values in the range 5–6 Å. But, a 12 Å *R*_g_ of the size modelled by Lou *et al.* would have a Guinier maximum *q* beginning at 0.108 Å^−1^, well within both *q* ranges for us to assess the size of any fullerene aggregates and model.

In contrast to SAXS, SANS is a much lower energy measurement. The energy of a 1 Å neutron is a little over 80 meV (cf. 12.4 keV for a 1 Å X-ray photon). The scattering/absorption of neutrons is also independent of the electron density of atoms. SANS data at the same *q* range (0.01–1 Å^−1^) as the data in figure 2 were collected, and the neutron transmission (electronic supplementary material, figure S4) through the solutions is ≥65% for 1 mm pathlength cuvettes (much greater than the X-ray transmission).

The SANS data are presented in figure 5 and show that in all cases, the scattering is flat and featureless. This indicates an absence of aggregates, or other structure, in the *q* range measured. We have verified that there is a sufficient neutron contrast in this solution system to be able to measure differences between the fullerene and the solvent (electronic supplementary material, tables S2 and S3). Consequently, we conclude that in all three of the solution cases considered (pure CB, pure DIO and CB with 3% DIO), the fullerene is molecularly dissolved.

We were unable to resolve the *R*_g_ for PC_71_BM in these solvents, due to a significant incoherent background component in these measurements (electronic supplementary material, table S2). To accurately measure the value for PC_71_BM, we have switched to the solvent carbon disulfide (CS_2_), which has been used previously to make OPV devices without the need for halogenated solvents [28]. Again using SANS and fitting a Guinier function, we measure an *R*_g_ value of 4.3 ± 0.1 Å (figure 6). This value compares well with the existing literature on C_70_, where previous SANS measurements on the fullerene C_70_ gave an *R*_g_ value of 4.13 ± 0.05 Å [29]. Another study again using CS_2_ demonstrated that SANS is sensitive to aggregates, in this instance for the fullerene C_60_, and the aggregates were attributed to differences in the preparation conditions, with 2 days dissolution time giving a solution free from aggregates [30].

**Figure 7.**
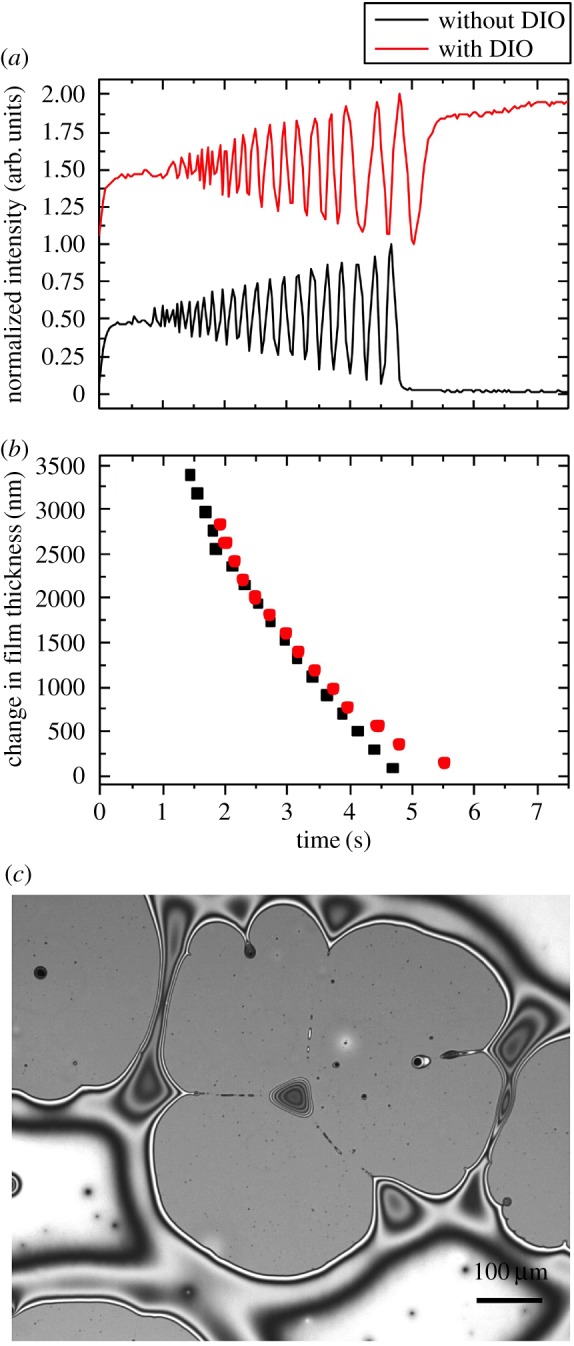
(*a*) Optical interference patterns showing the intensity changing as the film thins due to the process of evaporation. (*b*) The thickness change for the two solutions showing the divergence in the film drying dynamics. (*c*) Optical micrograph showing the surface of the spin-coated PC_71_BM thin film spun from CB with 3% v/v DIO.

We now shift our focus to what is happening to the PC_71_BM fullerene thin film layer as it dries. To understand the drying dynamics, *in situ* interferometry measurements were performed during spin coating [22,23]. Figure 7 shows the drying kinetics of the two fullerene solutions with the pure CB drying faster than when DIO is present. Ellipsometry was used to measure the final film thickness after spin coating. The solution without DIO gave a PC_71_BM film of 33 nm thickness, whereas the film cast from a solution in CB with 3% DIO was over three times thicker at 101 nm.

Both solutions produced continuous films during the spin casting process; however, we see how mobile the thin film cast from CB with 3% DIO is based on the observation of film breakup and dewetting structures [31] for the PC_71_BM layer, sometime after spin coating (approx. 1 h). This confirms that there is indeed enhanced molecular mobility of the PC_71_BM due to the retained DIO in the thin film after the spin coating process has finished and the majority of the chlorobenzene has been removed. Critically, immediately after casting, the film was continuous as was the layer cast from pure CB, which remained continuous and free from dewetting features. The PC_71_BM CB 3% DIO film will be somewhat more solvated than the case when a conjugated polymer is present, as the overall solid concentration will be nearly double the pure fullerene case. After 24 h, the dewetted film processed from CB plus 3% DIO had a similar film colour and thickness showing that the DIO had been removed by evaporation [16]. Dewetting-type structures would not be helpful in a working solar cell device as it will cause a reduction in the active contact area of the electrodes, also it will deplete the fullerene from the cathode interface, which has previously been shown to provide higher efficiency devices [32]. The concept of additive-enhanced mobility in these systems as being the principal origin for morphology improvements in OPVs is fully consistent with the dynamics studies in the literature [33]. Recent dynamic measurements have concluded that additives are important as they inhibit large-scale liquid–liquid phase separation of polymer and fullerene, preventing the formation of large polymer and fullerene domains [34].

## 4. Conclusion

**Figure 8.**
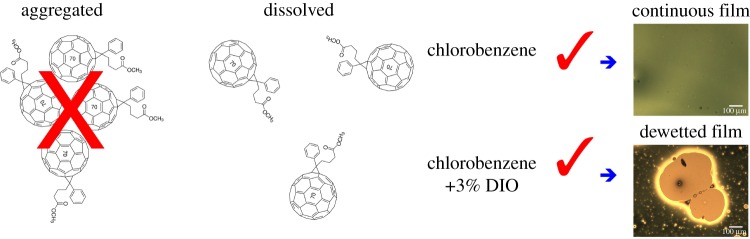
A summary of our conclusions based on our SANS and SAXS measurements. The optical images on the right show the difference in the final pure PC_71_BM film morphology; with the presence of DIO the film we see dewetting structures.

A prevalent assumption in the current literature is that the addition of DIO acts to improve performance in photovoltaic devices made from polymer–fullerene blend by dissolving or otherwise reducing the size of fullerene aggregates purported to exist in CB prior to spin coating. Unable to reproduce these observations, we carried out a full study aiming to understand the solution state of PC_71_BM in CB and CB with 3% DIO. Using both SAXS and SANS, we have shown that there is no change in the dissolution state of PC_71_BM upon the addition of DIO, and in fact, there is no aggregation of PCBM in solution *with or without* the additive, contrary to the analysis previously reported by Lou *et al.* [14]. Our SAXS and SANS measurements are in agreement and highlight the need for careful control of solvent backgrounds and absorption in such measurements, and that without careful control using either rapid exposures or flow, typical X-ray energies induce beam damage that appears consistent with unwanted aggregation. For both the solutions in CB only and CB with 3% DIO, we conclude that the PC_71_BM is molecularly dissolved. The combined use of the complementary techniques used in the present study casts considerable doubt upon this highly cited precedent in the literature [14]. Guinier analysis of the SAXS data in this work and the data extracted from the Lou *et al.* [14] paper are in agreement, showing that the fullerene is unchanged with and without DIO. Therefore, we conclude that the impact of the ‘additive’ DIO is that it resides in the film after the spin coating process has finished and allows enhanced molecular mobility of the DIO solvated fullerene on a much longer timescale than that of the rapid process of spin coating (figure 8). Formulation of polymer solar cell blends with suitable additives therefore relies on tailoring the additive to aid favourable morphology development during the extended drying phase [35]. Our results have important implications for device scientists and engineers attempting to maximize organic photovoltaic device quality and efficiency.

## Supplementary Material

Supporting Information

## Data accessibility

Data are available at the Dryad Digital Repository: (http://dx.doi.org/10.5061/dryad.553t5) [36]. The datasets supporting this article have been uploaded as part of the electronic supplementary material.
