# Supplementary material for: Does 1,8-diiodooctane affect the aggregation state of PC71BM in solution?
Source: R Soc Open Sci. 2018 Sep 12;5(9):180937. doi: 10.1098/rsos.180937 (PMC6170567; doi:10.1098/rsos.180937)
Supplement: Supporting Information [file rsos180937supp1.pdf]

Supporting Information

Does 1,8-Diodooctane (DIO) Affect the Aggregation State of $PC_{71}BM$ in Solution?

Gabriel Bernardo[§], Adam. L. Washington[†,#], Yiwei Zhang[§], Stephen. M. King[†], Daniel. T. W. Toolan[‡], Jonathan R. Howse[‡], Rajeev Dattani[¥], John Patrick A. Fairclough[#]* and Andrew J. Parnell[§]*

[§]Department of Physics and Astronomy, University of Sheffield, Sheffield S10 2TN, UK

[†]ISIS Pulsed Neutron and Muon Source Science and Technology Facilities Council, Rutherford Appleton Laboratory, Harwell Science and Innovation Campus, Didcot OX11 0QX, UK

[¥]European Synchrotron Radiation Facility (ESRF), Grenoble, France

[#]Department of Mechanical Engineering, The University of Sheffield, S3 7HQ, UK

[‡]Department of Chemical and Biological Engineering, The University of Sheffield, Sheffield S1 3JD, UK

**Sample**	**Standard Modelling I**	**Unified Fit**
$PC_{71}BM$ **without** DIO	235 ± 23 Å, and 11.5 ± 0.5 Å	170 ± 3, and 12.6 ± 0.3 Å
$PC_{71}BM$ with DIO	240 ± 35 Å, and 5.7 ± 1.1 Å	197 ± 2, and 5.2 ± 2.1 Å

Table 1. Comparison of the mean radii derived from model fitting two log normal distributions of spherical particles to SAXS data, taken directly from the supporting information of Lou *et al* [14].

**Material**	**x-ray SLD (@12.47 keV) [10-6Å-2]**	**x-ray SLD (@13 keV) [10-6Å-2]**	**x-ray SLD (@9.252 keV) [10-6Å-2]**	**Neutron SLD [10-6Å-2]**
DIO (diiodooctane) C8H16I2	14.52	14.51	14.53	0.12<br>(*incoherent 16.1*)
Chlorobenzene (C6H5Cl)	9.75	9.75	9.77	1.83<br>*(incoherent 12.32)*
dChlorobenzene (C6D5Cl)	9.73	9.73	(not used for the SAXS)	4.91<br>*(incoherent 2.35)*
$PC_{71}BM$ (C84H14O2)	12.90	12.90	12.92	4.43
DIO (3%v/v) CB solution	9.89	9.89	9.91	1.78
CS_2 solvent	10.78	10.78	10.89	1.23<br>*(incoherent 0.54)*

Table 2. Scattering length densities (SLD) for the materials that make up the system studied at the different x-ray energies and for neutrons

	**Δ SLD @ 12.47 keV**	**Δ SLD @ 13 keV**	**Δ neutron SLD**
CB / $PC_{71}BM$	3.15	3.15	3.08
DIO (3%v/v) CB / $PC_{71}BM$	3.01	3.01	3.13
dCB / $PC_{71}BM$	3.17	3.17	0.48
CS_2 / $PC_{71}BM$	2.12	2.12	3.68

Table 3. The difference in SLD that gives rise to the effective contrasts in SAXS and SANS.

Small angle X-ray scattering (SAXS)

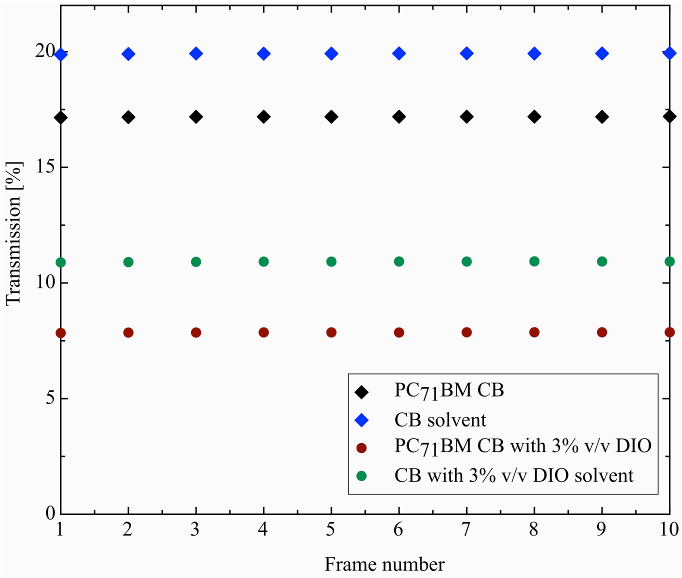

Figure S1. The transmission values for the samples and their backgrounds measured using SAXS, showing the ~ factor of 2 difference in the X-ray transmission with and without DIO all for the case of 15 mg/ml $PC_{71B}M$. The capillaries had the following diameters; CB solvent 1.34 mm, CB DIO solvent 1.40 mm, CB with PC71BM 1.48 mm, CB DIO PC71BM 1.58 mm.

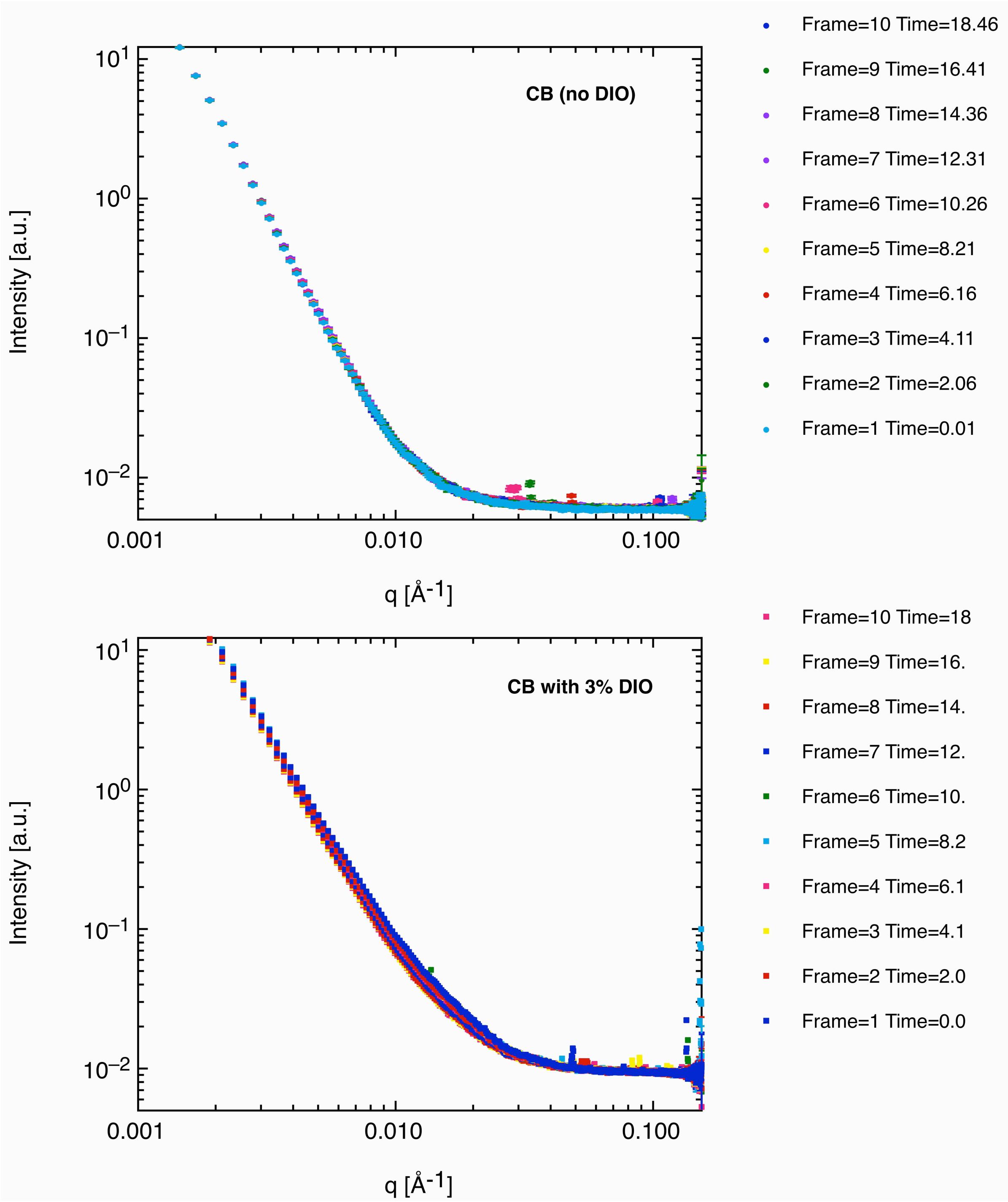

Figure S2. The respective solvents, CB and CB with 3% DIO 10 successive frames of X-ray exposure

Small angle neutron scattering (SANS)

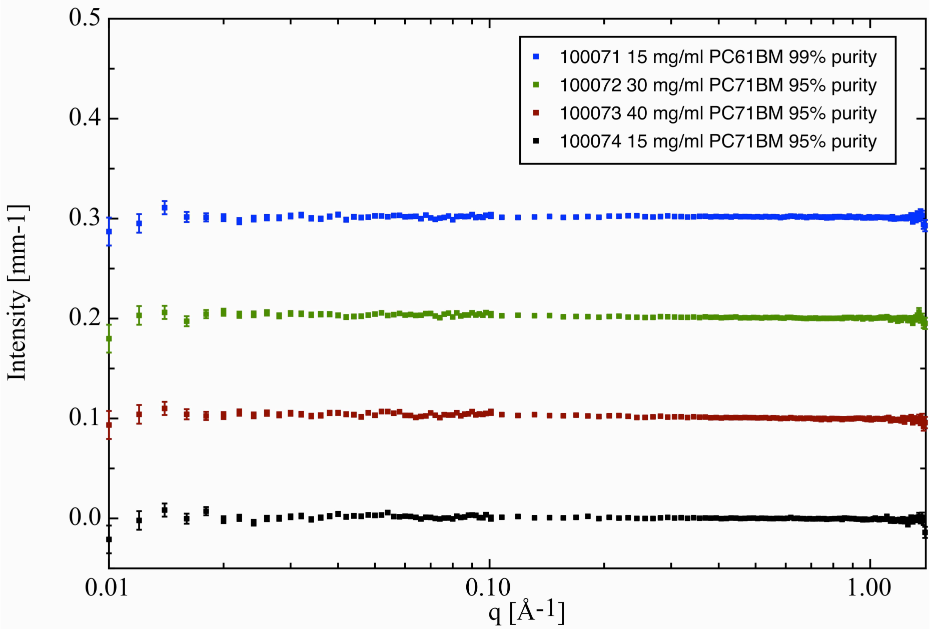

Figure S3. SANS data for higher fullerene concentration and repeat samples of fullerene in pure chlorobenzene, for $PC_{61}BM$ and $PC_{71}BM$ upto 40 mg/ml. The data are off set to enable detailed comparison, in reality they all sit at 0 mm^{-1}.

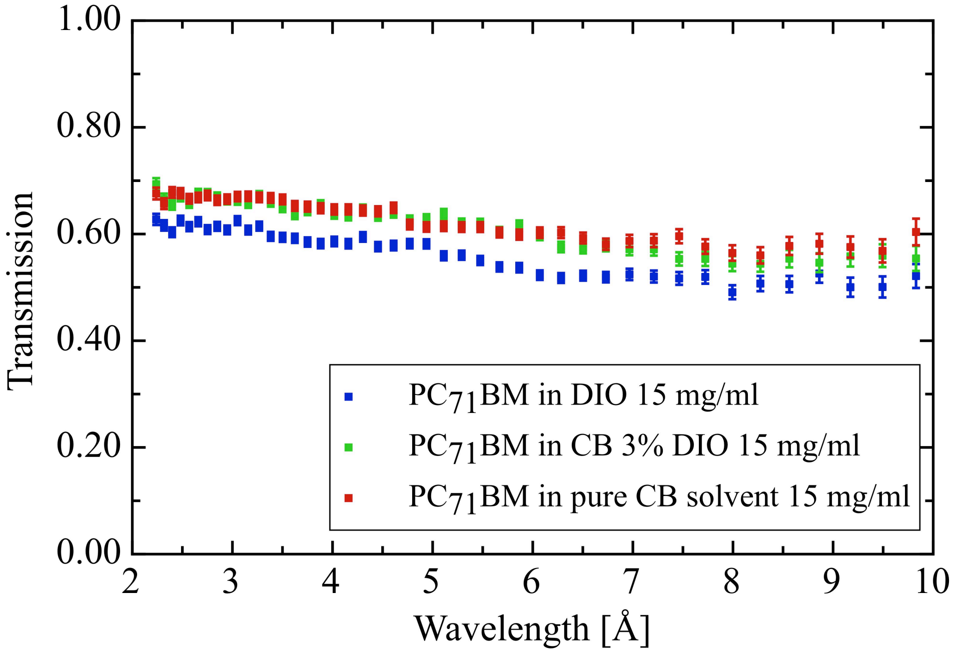

Figure S4. Neutron transmission for the three solutions studied, showing that well over half the incident neutron flux get through in all cases.

Ellipsometry

Spectroscopic ellipsometry (J.A. Woollam Co. M2000v) was used to measure the thickness of the spin coated $PC_{71}BM$ thin films. The ψ and Δ values were recorded over a wavelength range of 375−1000 nm. The film thickness was modeled with the software WVase using a Cauchy model over the optically transparent region (800 nm - 1000 nm) of the measured

wavelength range this gave thickness and refractive index.

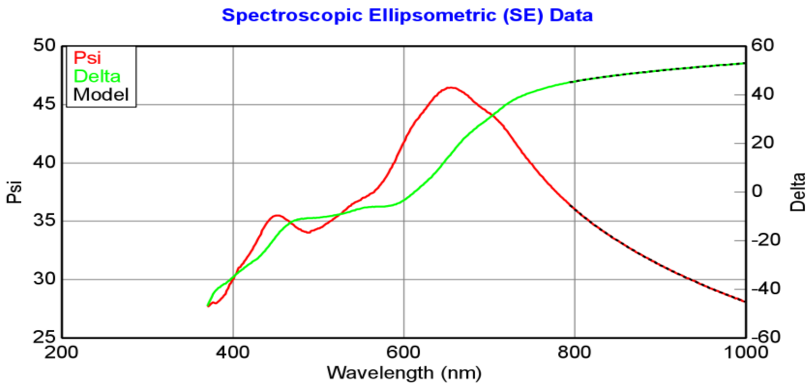

Figure S5. Ellipsometry data for $PC_{71}BM$ spincoated from pure chlorobenzene onto PEDOT:PSS.

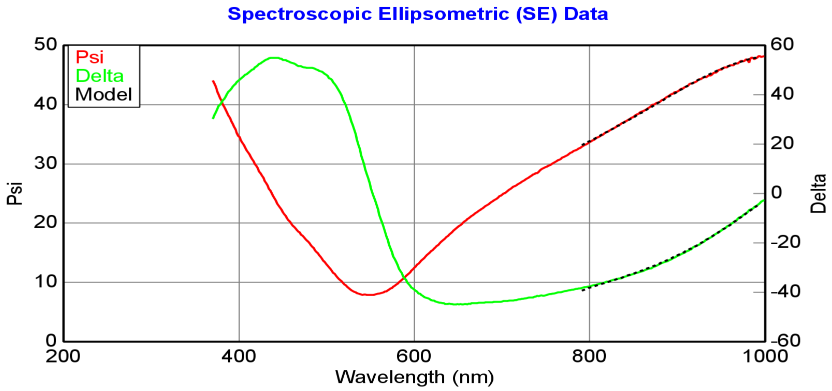

Figure S6. Ellipsometry data for $PC_{71}BM$ spincoated from chlorobenzene with 3% v/v DIO onto PEDOT:PSS.

Scanning probe microscopy

A Dimension 3100 machines was used in tapping mode to measure the surface topography of the two films. The tapping tips had a resonance near 275 kHz.

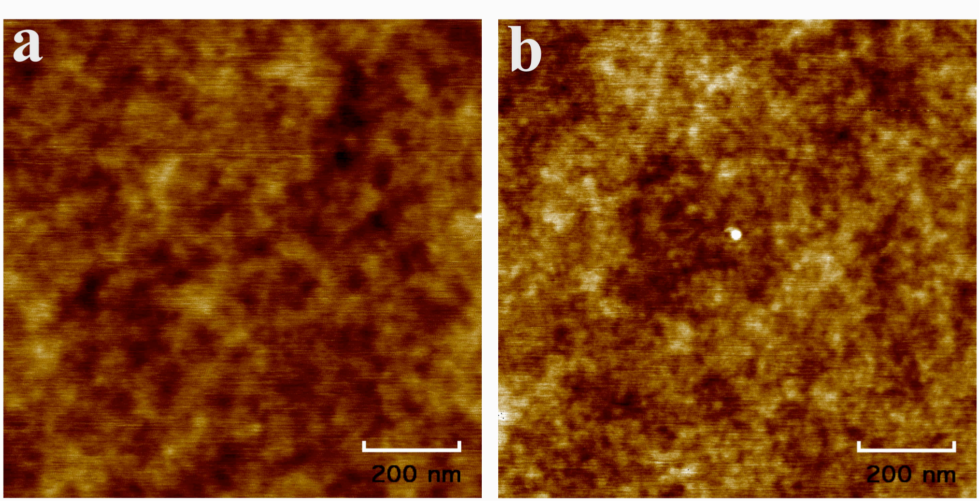

Figure S7. Scanning probe microscopy data for spin coated films of $PC_{71}BM$ in CB (a) and with 3% DIO (b).

The raw data was flattened. The rms roughness for the two films was measured as 0.28 nm for (a) and 0.24 nm for (b). the images were analyzed using the freely available program ImageSXM.

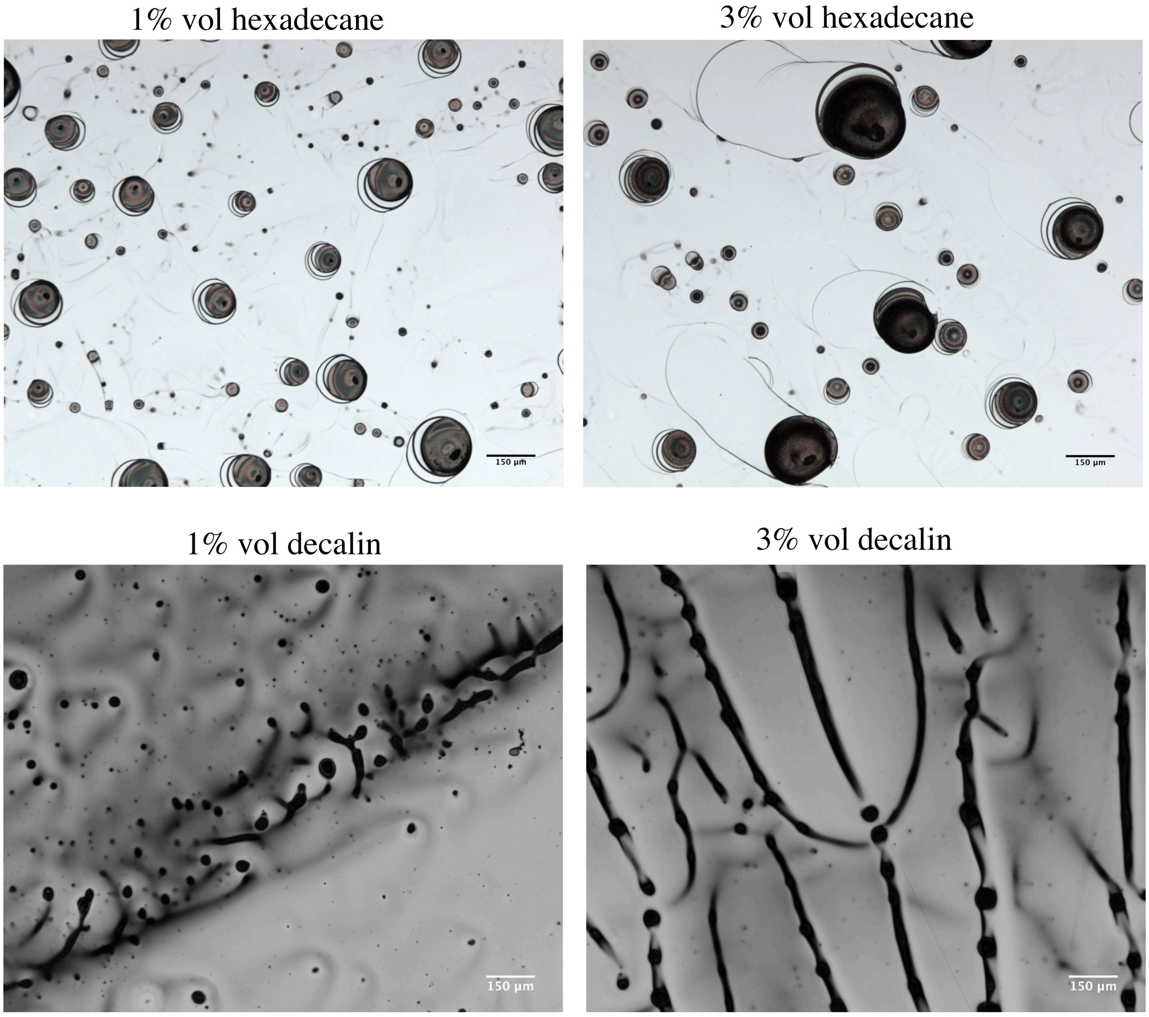

Figure S8. 15mg/ml PC71BM solutions with the addition of two further high boiling point solvents, namely decalin and hexadecane. The films start with a continuous film, which breaks up into the features seen in the images.

Optical images of $PC_{71}BM$ chlorobenzene solution processed films using the high boiling point solvent additives decalin and hexadecane in place of DIO, which respectively have boiling points of 187 ºC and 287 ºC. Both showed the same trend in behavior as was observed for the DIO, with an initial stable film which broke up and dewetted, albeit somewhat sooner than the case of DIO ~ seconds.

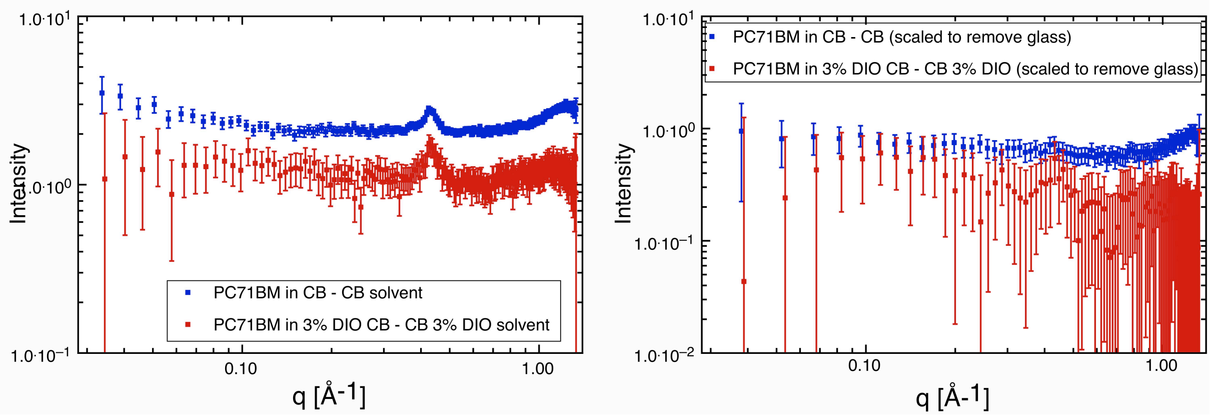

Figure S9. SAXS data measured in a flow cell to enable cleaner subtraction of the solvent background, with glass ~ 0.4 Å-1 and the chlorobenzene solvent ~ 1 $Å^{-1}$.

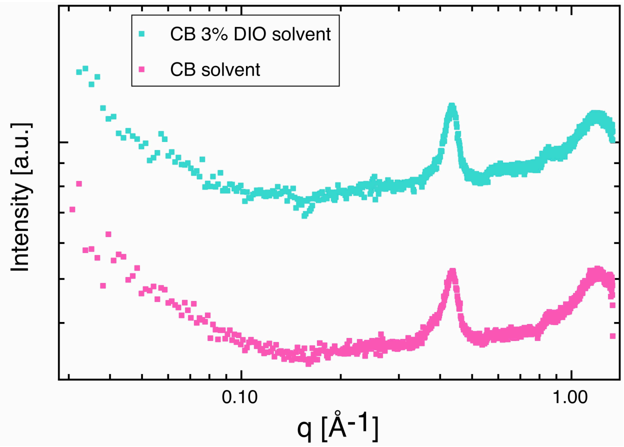

Figure S10. Solvent backgrounds showing the strong feature associated with the glass ~ 0.4$Å^{-1}$ and the chlorobenzene solvent ~ 1$Å^{-1}$.
